# Editorial: Strategies of virus persistence

**DOI:** 10.3389/fcimb.2026.1891851

**Published:** 2026-06-11

**Authors:** Magdalena Weidner-Glunde, Michael M. Nevels, Emma Poole

**Affiliations:** 1 Intercollegiate Faculty of Biotechnology of University of Gdańsk and Medical University of Gdańsk, Gdańsk, Poland; 2 University of St Andrews, St Andrews, United Kingdom; 3 University of Cambridge, Cambridge, United Kingdom

**Keywords:** coinfection, immune persistence, latency, reinfection, viral adaptation, viral circulation, virus persistence, virus quantification

This Research Topic compiles a broad spectrum of articles on virus persistence, collectively demonstrating that viral latency is not a passive state, but rather a dynamic driver of viral evolution, pathogenesis, and host immune manipulation. The featured studies span diverse subjects, ranging from the long-term persistence of the immune response following infection to targeted therapeutic strategies aimed at disrupting tumorigenic viruses. Furthermore, the collection highlights the critical need for single-cell analysis to reveal these complex viral mechanisms, concluding with a novel, optimized method designed to accurately quantify persistent viruses as they reactivate and transition into active replication.

One of the topics touched upon in the studies is how persistent infections act as active evolutionary engines (Nabieva et al.). Research on Severe Acute Respiratory Syndrome Coronavirus 2 (SARS-CoV-2) highlights how persistent infections in immunocompromised individuals, such as HIV-positive patients, function as evolutionary laboratories. By lingering long-term in environments like the gastrointestinal tract, the virus evades complete clearance, allowing it to accumulate mutations at an accelerated rate and potentially give rise to new variants of concern.

In contrast to the adaptive persistence of SARS-CoV-2, Liu et al. examine Human Papillomavirus (HPV). It is a classical persisting virus, whose genome is maintained as an episome, but its integration into the host DNA and consequent disruption of viral transcription triggers tumorigenesis. Liu et al. summarize current therapeutic efforts to excise the integrated viral genome using CRISPR/Cas gene-editing approaches. While early results in HPV-infected cells are highly promising, the authors emphasize that significant clinical challenges remain before this gene-editing therapy can be safely applied to patients.

Beyond viral evolution and oncogenesis, infection also profoundly reshapes the host’s immune landscape. This is evidenced by Xie et al.‘s study on Norovirus, which revealed a long-lasting immune imprinting effect driven by repeated exposures to the virus. The authors observed an unusual persistence of neutralizing antibodies, specifically genogroup-dependent memory for Norovirus GII, triggered by the reemergence of certain viral variants. The observed persistence of immunity to specific viral strains has implications for vaccine development.

Complementing these perspectives on persistence and immune modulation, Asaga et al. provide a population-level view of how repeated and overlapping viral exposures shape immune responses in endemic settings. Their large-scale surveillance study in Nigeria revealed extraordinarily high levels of arboviral exposure, with 86.8% of individuals showing evidence of recent infection and over 60% exhibiting coinfections involving Dengue, Chikungunya, Yellow fever, or Zika viruses. Importantly, the study demonstrates that coinfections are not immunologically neutral events but are associated with dose-dependent increases in pro-inflammatory and regulatory cytokines, indicating a compounded immune burden. Molecular analyses further revealed the co-circulation of multiple viral genotypes and lineages, including evidence of recent introductions, highlighting the dynamic nature of viral transmission networks. The strong association between infection prevalence and ecological as well as seasonal factors, particularly rainfall-driven vector expansion, underscores how environmental conditions sustain repeated viral exposure and shape long-term immune landscapes. Together, these findings reinforce the concept that persistence at the population level can emerge not only from classical latency, but also from continual reinfection and coinfection cycles that maintain immune activation and viral circulation.

Crucial to understanding these intricate interactions between host immunity and viral adaptation are high-resolution tools. Traditional methodologies often rely on bulk population averages, which can mask critical cellular behaviors. Zhang et al. review the new perspectives opened by single-cell RNA sequencing (scRNA-seq). This method, contrary to the methods based on analysis of a population average, allows to more fully understand the viral persistence. It enables the identification of vary rare cells carrying persistent viruses, such as Human Cytomegalovirus (HCMV). The authors highlight the possibility of using scRNA-seq to characterize infection-induced novel cell types. This is crucial for understanding why some patients clear a virus while others, like the HIV-positive patient described by Nabieva et al., develop a chronic, evolving infection. The scRNA-seq method enables characterisation of the heterogeneity of cell population, allowing separation of subpopulations of cells that respond differently to virus infection. This method also allows characterisation of virus-specific host cell gene expression changes, defines viral target cells and enables clarification of *in vivo* virus distribution.

Methods to quantify lytic viruses are required for studies of experimental latency and when a persistent virus transitions from latency to active replication. Addressing this need, Kelnhofer-Millevolte et al. developed an optimized method for the quantification of HCMV particles. They test the method across multiple imaging platforms, including confocal microscopy, a Bio-Rad imaging system, and even smartphone cameras, demonstrating that while high-end microscopy gives the best sensitivity, simpler and widely accessible tools still provide reliable results. The authors show that their approach can accurately detect and quantify viral plaque numbers and sizes with high precision and sensitivity, reducing the need for time-consuming manual counting. The study provides a practical, adaptable, and cost-effective workflow for HCMV quantification that may be extended to other viruses.

## Conclusion

Ultimately, this Research Topic underscores that understanding viral persistence requires a multifaceted approach that bridges basic virology, immunology, and innovative technology. Whether documenting the accelerated mutation rates of SARS-CoV-2 in immunocompromised niches, designing CRISPR therapies to disrupt tumor-causing HPV integration, or mapping out immune modulation by Norovirus and arboviruses, persistence remains a formidable clinical hurdle. However, by leveraging advanced investigative tools, such as scRNA-seq and refined particle quantification methods, the scientific community is uniquely positioned to translate these mechanistic insights into targeted therapeutic interventions and robust vaccine strategies.

